# Experimental and Theoretical Studies on the Corrosion Inhibition of Carbon Steel by Two Indazole Derivatives in HCl Medium

**DOI:** 10.3390/ma12081339

**Published:** 2019-04-24

**Authors:** Shenying Xu, Shengtao Zhang, Lei Guo, Li Feng, Bochuan Tan

**Affiliations:** 1 School of Chemistry and Chemical Engineering, Chongqing University, Chongqing 400044, China; 20111802011@cqu.edu.cn (S.X.); 20151802007@cqu.edu.cn (L.F.); 20161802006@cqu.edu.cn (B.T.); 2 School of Chemistry and Chemical Engineering, Yibin University, Yibin 644000, China; 3 School of Material and Chemical Engineering, Tongren University, Tongren 554300, China

**Keywords:** corrosion inhibitor, carbon steel, indazole derivatives, electrochemistry, DFT

## Abstract

In this work, two indazole derivatives, namely 5-aminoindazole (AIA) and 5-nitroindazole (NIA), were investigated as corrosion inhibitors for carbon steel in 1 M HCl solution by experimental and density functional theory (DFT) methods. The electrochemical results indicate that the inhibition ability follows the order of AIA > NIA, which is due to the stronger electron-donating effect of –NH_2_ of the AIA group than the –NO_2_ group of NIA. Besides, the frontier orbital theory shows that the AIA exhibits higher reaction activity than NIA, and a more negative adsorption energy for AIA was also obtained, which is consistent with the analysis of the electrochemical measurements. We draw the conclusion that the electron-donating effect makes it easier for AIA to donate electrons to iron atoms to form a stronger protective layer than NIA.

## 1. Introduction

**Figure 1:**
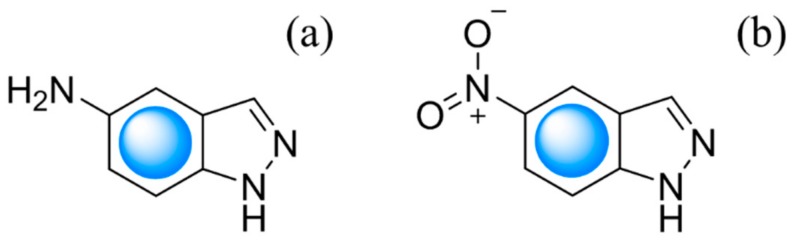
Molecular structures of indazole derivatives, (**a**) 5-aminoindazole; (**b**) 5-nitroindazole.

Carbon steels are extensively utilized as structural materials in plenty of industrial fields, especially in corrosive environments. In view of this, some protective strategies have been employed to protect steel from corrosion. The addition of corrosion inhibitors has been proved to be an easy and highly effective way to achieve this [1,2,3]. Organic compounds containing heteroatoms such as N, P, S or O have been used as excellent inhibitors due to their strong electron-donating ability [4,5,6]. Additionally, due to their high adsorption capacity, indazole derivatives have attracted the attention of several researchers [7,8]. Our previous work revealed that indazole can absorb onto copper surfaces and exhibits a favorable inhibition efficiency for copper corrosion in a 3.0 wt.% NaCl solution [7]. Furthermore, the influence of active adsorption, which centers on the inhibition effectiveness of IA-based inhibitors, was also researched in aggressive solutions [9]. Potentially, it is important to explore the effects of functional groups of indazole-based inhibitors on inhibition effectiveness. Herein, 5-aminoindazole (AIA) and 5-nitroindazole (NIA), as shown in Figure 1, were discovered for the first time to be effective corrosion inhibitors for carbon steel in hydrochloric acid (HCl) solution.

Specifically, this work focused on the corrosion inhibition of AIA and NIA for Q235 carbon steel in 1 M hydrochloric acid solution by using multiple techniques including electrochemical measurements, scanning electron microscopy (SEM) and DFT calculations. On account of the experimental and theoretical results, the difference of the inhibition mechanisms between AIA and NIA molecules was revealed, which would provide some reference functions to develop more efficient inhibitors for corrosion protection.

## 2. Experimental Section

### 2.1. Material and Samples Preparation

The tested inhibitors were AIA and NIA (Aladdin Company, Shanghai, China). The chemical composition of Q235 carbon steel (JIS G3101) was 0.17% C, 0.47% Mn, 0.26% Si, 0.017% S, 0.0048% P and Fe. The specimens for electrochemical measurements were sealed in ethoxyline resin with a 1 cm^2^ area exposed as working area. Prior to experiments, all specimens were polished by emery paper from 400 to 1200 grit. Then, samples were cleaned by ethanol and distilled water and dried at room temperature. Analytical reagent grade 37.5% water-diluted HCl was used as the corrosive medium (1 M HCl). 

### 2.2. Electrochemical Measurements

A three-electrode cell was used to do the electrochemical experiments, and all tests were performed in a CHI660B CHI660B electrochemical workstation (Chinstruments, Shanghai, China). Q235 carbon steel, a platinum plate of 1.5 × 1.5 cm^2^, and a saturated calomel electrode (SCE) with a Luggin capillary were the working electrode, counter electrode, and reference electrode, respectively. To obtain a steady open circuit potential (OCP), the electrodes were immersed into the corrosive solution for one hour before tests. The potentiodynamic polarization curves were obtained with the potential range from −250 mV to 250 mV vs. OCP at the scan rate of 0.2 mV s^−1^. Electrochemical impedance spectroscopy (EIS) experiments were performed in a frequency range from 10^−2^ Hz to 10^5^ Hz with amplitude of 5 mV at OCP (−0.45 V to −0.49 V). Z-view software was employed to fit the experimental data by an appropriate equivalent circuit.

### 2.3. Scanning Electron Microscopy

The surface morphologies of Q235 carbon steel specimens were captured with scanning electron microscopy (SEM, Joel-6490LV, Tokyo, Japan) with an accelerating voltage of 20 kV. Before SEM characterization, the samples were immersed for six hours in 1 M HCl with and without AIA or NIA inhibitor. 

### 2.4. Theoretical Simulations

A Gaussian 03W program was employed for quantum chemical calculations based on DFT. The geometry optimized structures of AIA and NIA were obtained through the use of the B3LYP function with a 6-311++G(d, p) basis set. During this process, no imaginary frequency was confirmed and the structures were in their lowest-energy state. Furthermore, DFT was used to calculate the Mulliken charge, dipole moment (*μ*) and frontier molecular orbitals including the energy of the highest occupied molecular orbital (*E*_HOMO_), the energy of the lowest unoccupied molecular orbital (*E*_LUMO_) and energy gap (Δ*E* = *E*_LUMO_ − *E*_HOMO_).

The interactions between AIA or NIA and the Fe (110) surface were modeled in a simulation box (12.4 Å × 9.9 Å × 24.1 Å) with periodic boundary conditions by the Dmol^3^ program of Material Studio software (BIOVIA, USA). A 4-layer 5 × 5 supercell (the lower two layers were constrained) with 20 Å vacuum slab was used to simulate bulk metal. DFT calculations were treated within the generalized gradient approximation (GGA) function of Perdew–Burke–Ernzerhof (PBE) and the double numerical basis set with polarization functions on hydrogen atoms (DNP). DFT semi-core pseudopots (DSPPs) were used for Fe treatment. The displacement convergence, gradient, and tolerances of energy were 5 × 10^−3^ Å, 2 × 10^−3^ Ha·Å^−1^, and 1 × 10^−5^ Ha, respectively. The interaction energy (*E*_Fe-inhibitor_) between the Fe (110) surface and inhibitor obeyed [10,11,12]

*E*_Fe-inhibitor_ = *E*_Total_ − *E*_Fe_ − *E*_inhibitor_ (1)

where *E*_Total_ is the energy of Fe(110) surface and the adsorbed inhibitor molecule, *E*_inhibitor_ is the energy of the isolated inhibitor molecule and *E*_Fe_ is the energy of the steel surface, respectively.

## 3. Results and Discussion

### 3.1. Electrochemical Impedance Spectroscopy (EIS) Measurements 

**Figure 2:**
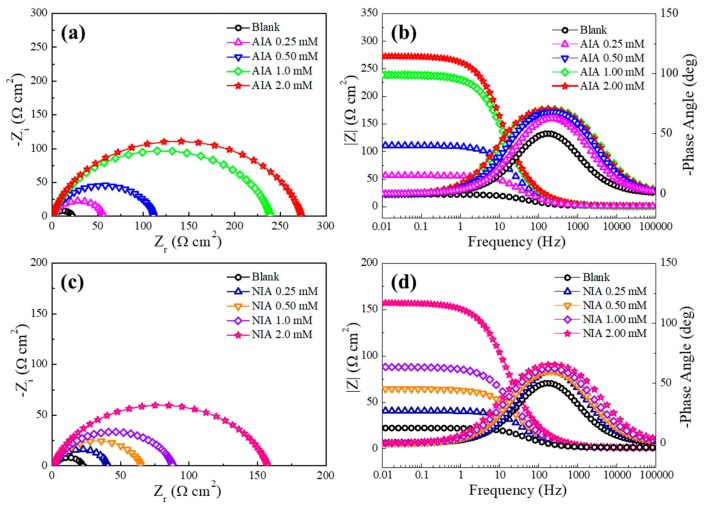
The plots of Nyquist and Bode for carbon steel in 1 M HCl with and without different concentrations of 5-aminoindazole (AIA) and 5-nitroindazole (NIA) at 298 K. AIA: (**a**,**b**); NIA: (**c**,**d**).

Figure 2 shows the electrochemical impedance spectra plots of AIA and NIA, respectively. In Figure 2a,c, it can be seen that the radius of the capacitive resistance arc increased with the growing concentration of inhibitors, which indicates that the protective layer was formed on the steel surface by the adsorption of AIA or NIA and that the corrosion inhibition efficiency of AIA is better than NIA. In addition, the impedance spectrum exhibits a squashed semicircle, which is caused by the formation of a protective layer on the steel surface. Figure 2b,d is Bode plots in the presence and absence of AIA and NIA, respectively. The impedance values and phase angle values increase with the growing concentration of inhibitors. In addition, a time constant can be found in the phase angle, usually due to the relaxation effect of the corrosion inhibitor molecule adsorption [2,13].

**Figure 3:**
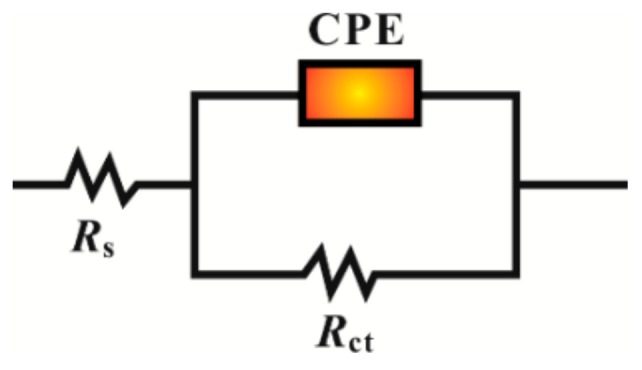
Electrical equivalent circuit used to fit the electrochemical impedance spectroscopy (EIS) experimental data. CPE: constant phase element.

The equivalent circuit (Figure 3) is used to fit the impedance spectrum data, and the fitting data are shown in Table 1. In Figure 3, *R*_s_ is the solution resistance, *R*_ct_ is the charge transfer resistance, and CPE is a constant phase element; the impedance of the CPE is expressed as follows [14,15]:

(2) $$Z_{\mathrm{CPE}}=\frac{1}{Y_{0}{\left(j\omega \right)}^{n}}$$

The double-layer capacitance (*C*_dl_) can be calculated from CPE parameter values *Y*_0_ and *n* by the following expression [16]:

(3) Cdl=Y0ωn−1sin(nπ2)

where *Y*_0_ is the CPE constant, *n* is the phase shift, which can be explained as a degree of surface inhomogeneity, *j* is the imaginary unit and *ω* is the angular frequency. The inhibition efficiency *η*_EIS_ can be expressed by the following equation [17,18,19]:

(4) $$\eta_{\mathrm{EIS}}=(\frac{R_{\mathrm{ct}}-R_{\mathrm{ct}}^{0}}{R_{\mathrm{ct}}})\times 100$$

where *R*_ct_ and *R*_ct,0_ are the charge transfer resistances with and without AIA and NIA, respectively. In Table 1, we can see that as the concentration of the corrosion inhibitor increases, the value of the *R*_ct_ becomes larger, and the value of *η*_EIS_ also increases simultaneously. When the concentration of the inhibitor was 2 mM, the *R*_ct_ values of AIA and NIA are 238 and 156.2 Ω cm^2^, respectively. According to the Helmholtz model formula, the value of double layer capacitance (*C*_dl_) can be expressed as [20,21]

(5) $$C_{\mathrm{dl}}=\frac{\varepsilon^{0}\varepsilon }{d}S$$

where *ε*^0^ is the dielectric constant of air and *ε* is the local dielectric constant. *S* is the surface area of the working electrode, and *d* is the surface film thickness. Compared with water molecules, the molecular volume of AIA and NIA is significantly larger, and their dielectric constant is smaller than that of water molecules. Therefore, with increasing concentrations of AIA or NIA, the two investigated inhibitors replace the water molecules on the surface of carbon steel continuously, and the value of *C*_dl_ decreases. Hence, the smaller the *C*_dl_, the denser the protective film formed on the surface of the carbon steel by AIA and NIA.

### 3.2. Potentiodynamic Polarization Measurements

**Figure 4:**
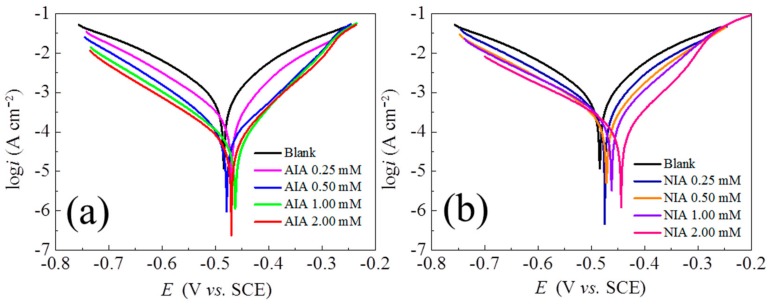
Anodic and cathodic polarization curves for carbon steel in 1 M HCl with various concentrations of (**a**) AIA and (**b**) NIA at 298 K. SCE: saturated calomel electrode.

Figure 4 shows the electrodynamic polarization curves of carbon steel at 298 K in 1 M HCl solutions with various concentrations of NIA and AIA. The relevant data (in Table 2) are obtained by the extrapolation method. The inhibition efficiency *η*_P_ is calculated by the following equation [22,23,24]:

(6) $$\eta_{p}=(\frac{i_{\mathrm{corr}}^{0}-i_{\mathrm{corr}}}{i_{\mathrm{corr}}^{0}})\times 100$$

where *i*^0^_corr_ and *i*_corr_ are the corrosion current density in 1 M HCl solutions without and with the two investigated inhibitors, respectively. 

It can be seen in Figure 4 that, compared with the values in blank solution, the corrosion potential (*E*_corr_) and corrosion current density (*I*_corr_) of the polarization curves change obviously with the addition of the two investigated inhibitors. With the addition of AIA and NIA, the *I*_corr_ decreases and the *E*_corr_ moves in a positive direction, illustrating that the corrosion reaction is effectively controlled. Clearly, the investigated inhibitors not only reduced the corrosion of the cathode but also reduced the corrosion of the anode. In addition, the shapes of polarization curves are parallel with the increase of concentration for the two investigated inhibitors, indicating that the action mechanism is same under different concentrations of inhibitors. Generally, it is considered as a cathodic inhibitor when the potential change exceeds 85 mV, while it is a mixed inhibitor when the potential change is less than 85 mV [25,26]. From Table 2, the changed values of *E*_corr_ for the two inhibitors are less than 85 mV, suggesting that AIA and NIA are mixed corrosion inhibitors. The changed values of *β*_a_ and *β*_c_ also reflect the cathodic and anodic corrosion rates being retarded by the studied inhibitors. Furthermore, the inhibition efficiency (*η*_p_) is also improved with increasing concentrations of the two inhibitors, and the inhibition ability follows the order AIA > NIA, which may be due to the stronger electron-donating effect of –NH_2_ than the –NO_2_ [27,28]. The electron-donating effect of the –NH_2_ makes the electron cloud density of the whole AIA molecule larger than the –NO_2_ of NIA, which leads to AIA finding it easier to give electrons to iron atoms and having a better protective effect than NIA. 

### 3.3. Morphology Analysis

**Figure 5:**
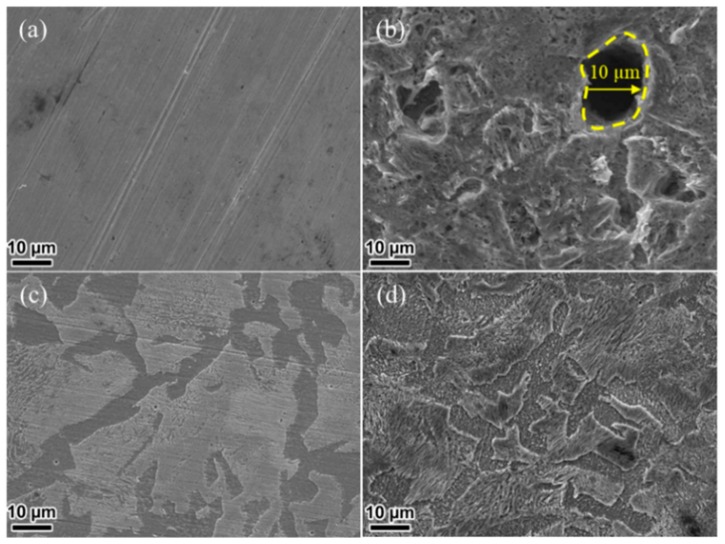
SEM images of (**a**) fresh carbon steel and carbon steel immersed in 1 M HCl solution (**b**) without and with 2 mM (**c**) AIA or (**d**) NIA.

To obviously display the inhibition differences between AIA and NIA inhibitors, the morphologies of untreated and treated carbon steel were studied. The obtained images are shown in Figure 5. The surface of fresh carbon steel is smooth, while large holes and cracks with a size of 10 µm were observed after immersion in 1 M HCl (Figure 5a,b). The pitting morphology is formed due to the elimination rate of corrosion products slower than the reaction rate between Cl^−^ and Fe [29,30]. Besides, some small holes and cracks still appeared on the steel surface even after 2 mM NIA was added into the HCl solution (Figure 5d). However, a flat surface was achieved with the addition of 2 mM AIA (Figure 5c); meanwhile the steel surface was covered with the absorbed AIA. Hence, the AIA shows prior inhibition performance than NIA. These results are in good agreement with electrochemical measurements.

### 3.4. Computational Study

**Figure 6:**
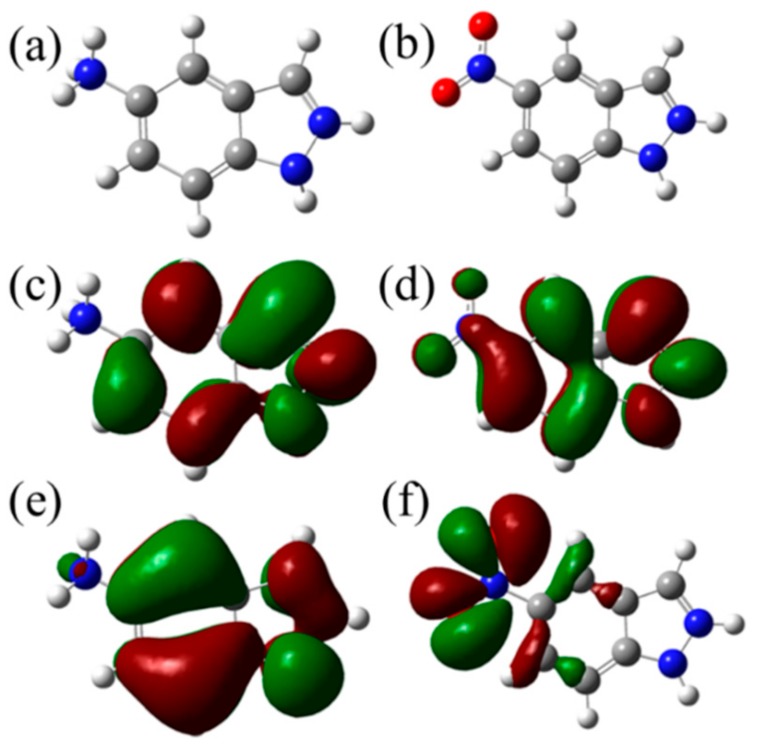
(**a**,**b**) Optimized geometric structures, (**c**,**d**) LUMO orbitals and (**e**,**f**) HOMO orbitals of AIA and NIA inhibitors.

Computational simulation is an effective way to explain reaction mechanisms. In this work, DFT calculations were used to reveal the adsorption and inhibition performances of AIA and NIA molecules. By comparison, the acidity coefficient (p*K*_a_) of both AIA and NIA displayed higher values than the pH of 1M HCl medium, proving the existence of the protonated molecule. Specifically, there are two p*K*_a_ (1.89 and 3.42) for AIA, indicating the existing form of AIA-2H^+^ in HCl solution. Instead, there is only one p*K*_a_ for NIA, indicating the existence of NIA-H^+^. The optimized geometry structure and frontier molecular orbitals (the highest occupied molecular orbital (HOMO) and the lowest unoccupied molecular orbital (LUMO)) of AIA-2H^+^ and NIA-H^+^ are shown in Figure 6, and Table 3 shows the energy of HOMO (*E*_HOMO_) and LUMO (*E*_LUMO_). It is generally known that HOMO is related to the ability of a molecule to donate electrons, and a higher *E*_HOMO_ value shows a stronger electron-donating ability [31,32]. By contrast, LUMO is associated with the electron-accepting ability of a molecule, and a lower value of *E*_LUMO_ represents a strong electron-accepting ability [33]. As can be seen from Figure 6c,d, the LUMO of both AIA-2H^+^ and NIA-H^+^ is distributed uniformly around the whole molecule. Conversely, in comparison with AIA-2H^+^ (Figure 6f), the HOMO of NIA-H^+^ was mainly delocalized around the nitro-substituent (Figure 6e), indicating that the electron-donating ability of NIA mainly comes from the nitro-substituent. According to the frontier orbitals, both AIA and NIA tend to interact with the steel surface through the π bond of rings in the way of parallel adsorption configuration.

In addition, the HOMO–LUMO gap (Δ*E*) is an important parameter to evaluate the stability of inhibitors, and the lower value of Δ*E* indicates that the inhibitor molecule could more easily adsorb on the metal surface [34,35]. As shown in Table 3, both AIA-2H^+^ and NIA-H^+^ have lower values of Δ*E* (3.4 eV and 4.6 eV, respectively), resulting in their strong ability to accept electrons from the d-orbital of steel as well as the high stability of the [Fe-inhibitor] complexes; namely, the AIA exhibited higher reaction activity than NIA. At the same time, the dipole–dipole (*μ*) interaction between the inhibitor and metal surface could improve the inhibition efficiency [36]. Herein, the fact that *μ*_NIA_ is about three times *μ*_AIA_ indicates that AIA exhibits more appropriate adsorption between the AIA and metal surface than NIA.

The ionization potential (*I* = −*E*_HOMO_) and electron affinity (*A* = −*E*_LUMO_) could be used to derive the electronegativity (*χ*) and global hardness (*γ*). The fraction of the electron transfer (Δ*N*) between the inhibitor molecules and Fe surface is given by following equation [37,38,39]:

(7) $$\Delta N=\frac{\chi_{\mathrm{Fe}}-\chi_{\mathrm{inh}}}{2\left(\gamma_{\mathrm{Fe}}+\gamma_{\mathrm{inh}}\right)}$$

where *χ*_Fe_ and *γ*_Fe_ are the absolute electronegativity and hardness of the Fe atom; and *χ_i_*_nh_ and *γ*_inh_ are the absolute electronegativity and hardness of the inhibitor molecules. A theoretical *χ*_Fe_ value of bulk Fe is 7 eV/mol, whereas *γ*_Fe_ is almost zero. *χ_i_*_nh_ and *γ*_inh_ are related to *I* and *A* [40,41].

(8) $$\chi =\frac{I+A}{2}$$

(9) $$\gamma =\frac{I-A}{2}$$

The direction of electron transfer is manifested by positive or negative Δ*N* values [42]. From Table 3, both AIA and NIA are electron acceptors. It is noteworthy that the magnitude of Δ*N′s* absolute value is not connected with inhibition efficiency.

**Figure 7:**
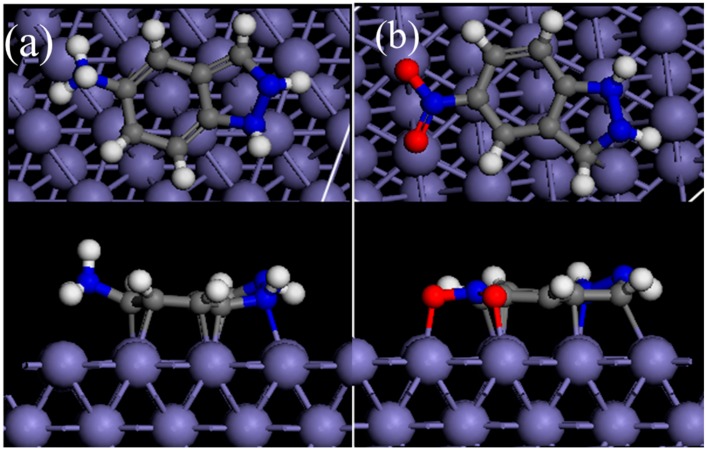
Stable adsorption configurations (side and top view) of (**a**) AIA-2H^+^ and (**b**) NIA-H^+^ molecules on the Fe(110) surface.

AIA-2H^+^ and NIA-H^+^ were placed in a simulation box parallel with or perpendicular to the Fe(110) surface. The simulation results showed that both AIA-2H^+^ and NIA-H^+^ tended to adsorb in parallel on the Fe(110) surface, as shown in Figure 7. Namely, the indazole and aromatic rings were the adsorption sites, which was in agreement with previous reports. Besides, all the hydrogen atoms upturning after adsorption may be due to the hybridization between Fe and heavy atoms. The AIA molecule is possibly a more efficient inhibitor because of its more negative adsorption energy (−4.65 eV) than NIA (−4.05 eV). This is consistent with the analysis of the electrochemical measurements. 

**Figure 8:**
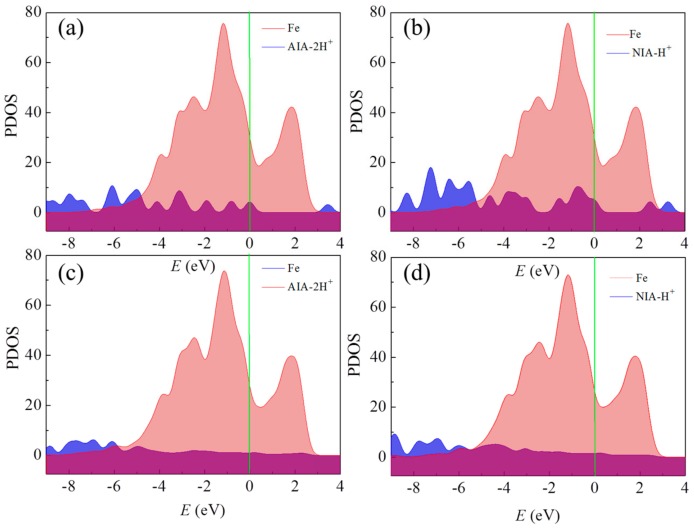
Density states projected of (**a**,**c**) AIA-2H^+^ and (**b**,**d**) NIA-H^+^ molecules before and after adsorbing on the Fe(110) surface.

Figure 8 shows the projected density states of AIA-2H^+^ and NIA-H^+^ before and after adsorbing on the Fe(110) surface. By comparing these with the isolate inhibitors, the p orbitals of the adsorbed inhibitors almost disapear, revealing the strong interaction between AIA or NIA and the Fe(110) surface [43]. This is consistant with the inhibition efficencies obtained by experiments.

## 4. Conclusions

In this study, two indazole derivatives, AIA and NIA, were proved to be excellent corrosion inhibitors for carbon steel in 1 M HCl. The inhibition performance was tested by electrochemical methods. Theoretical calculations were also performed to reveal the inhibition mechanism of AIA and NIA. The detailed results are as follows:

(1) The results of electrochemical tests indicated that AIA and NIA are efficient inhibitors for carbon steel in 1M HCl. The inhibition efficiency increased with increasing concentrations of the inhibitors, and the optimal concentration of AIA and NIA is 2 mM. By comparison, the AIA exhibits better inhibition performance than NIA.
(2) The values of the charge transfer resistance increased in the presence of AIA and NIA in EIS tests, indicating that they can protect steel from corrosion by forming a robust protective film. Additionally, the Tafel plots illustrated that both are mixed-type inhibitors.
(3) The results of theoretical calculations explained that the protective effect was due to the electrostatic forces between the AIA-2H^+^ (or NIA-H^+^) and electronegative surface.

## Figures and Tables

**Table 1:** Impedance data for Q235 steel in 1 M HCl with various concentrations of NIA and AIA at 298 K.

Inhibitor	*C*_inh_ (mM)	*R*_s_ (Ω cm^2^)	*R*_ct_ (Ω cm^2^)	CPE		*C*_dl_	*η*_EIS_ (%)
				*Y*_0_ × 10^−6^ (S s^n^ cm^−2^) *n*		(μF cm^−2^)	
Blank	/	1.43	20.1	504.6	0.85	215.4	/
AIA	0.25	1.26	55.3	214.5	0.85	188.8	62.1
	0.50	1.11	109.9	159.6	0.88	140.4	80.9
	1.00	1.22	238.0	120.3	0.87	104.7	91.2
	2.00	1.36	271.6	113.4	0.87	98.7	92.3
NIA	0.25	1.23	39.6	280.4	0.88	246.8	47.1
	0.50	1.34	63.4	265	0.84	222.6	66.9
	1.00	1.22	86.9	247.4	0.84	207.8	75.9
	2.00	1.14	156.2	185.9	0.83	154.3	86.6

**Table 2:** Relevant parameters for Q235 steel in 1 M HCl solution in the absence and presence of different concentrations of AIA and NIA at 298 K from polarization curves.

Inhibitor	*C*_inh_ (mM)	*E*_corr_ (V vs. SCE)	*β*_c_ (mV dec^−1^)	*β*_a_ (mV dec^−1^)	*I*_corr_ (μA cm^−2^)	*η*_p_ (%)
Blank	/	−0.48	119.4	95.3	754.4	/
AIA	0.25	−0.47	112.0	73.9	231.1	69.4
	0.50	−0.48	70.38	78.7	72.2	90.4
	1.00	−0.47	115.3	78.7	59.8	92.1
	2.00	−0.48	81.95	82.7	34.5	95.4
NIA	0.25	−0.48	122.6	82.8	363.7	51.8
	0.50	−0.47	127.7	85.6	248.8	67.0
	1.00	−0.46	132.8	85.8	239.9	68.2
	2.00	−0.45	107.2	74.6	117.4	84.4

**Table 3:** Quantum chemical parameters for AIA and NIA by using the B3LYP/6-311 + + G(d,p) method.

Inhibitor	*E*_HOMO_ (eV)	*E*_LUMO_ (eV)	Δ*E* (eV)	*μ* (Debye)	*I* (eV)	*A* (eV)	*χ* (eV)	*γ* (eV)	Δ*N*
AIA	−9.6	−6.2	3.4	5.4	9.6	6.2	7.9	1.7	−0.25
NIA	−11.7	−7.1	4.6	14.3	11.7	7.1	9.4	2.3	−0.53
